# Evaluation of Antibiotics Residues in Milk and Meat Using Different Analytical Methods

**DOI:** 10.1155/2023/4380261

**Published:** 2023-06-30

**Authors:** Melaku Getahun, Rahel Belete Abebe, Ashenafi Kibret Sendekie, Alem Endeshaw Woldeyohanis, Asmamaw Emagn Kasahun

**Affiliations:** ^1^Department of Veterinary Pharmacy, College of Veterinary Medicine and Animal Sciences, University of Gondar, P.O. Box 196, Gondar, Ethiopia; ^2^Department of Clinical Pharmacy, School of Pharmacy, College of Medicine and Health Sciences, University of Gondar, P.O. Box 196, Gondar, Ethiopia; ^3^Department of Pharmaceutics and Social Pharmacy, School of Pharmacy, College of Medicine and Health Sciences, University of Gondar, P.O. Box 196, Gondar, Ethiopia

## Abstract

Veterinary drugs are pharmacologically and biologically active chemical agents. At present, veterinary drugs are extensively used to prevent and treat animal diseases, to promote animal growth, and to improve the conversion rate of feed. However, the use of veterinary drugs in food-producing animals may leave residues of the parent compounds and/or their metabolites in food products resulting in harmful effects on humans. To ensure food safety, sensitive and effective analytical methods have been developing rapidly. This review describes sample extraction and cleanup methods, and different analytical techniques are used for the determination of veterinary drug residues in milk and meat. Sample extraction methods, such as solvent extraction, liquid-liquid extraction, and cleanup methods such as dispersive solid-phase extraction and immunoaffinity chromatography, were summarized. Different types of analytical methods such as microbial, immunological, biosensor, thin layer chromatography, high-performance liquid chromatography, and liquid chromatography–tandem mass spectrometry were discussed for the analysis of veterinary drug residues in animal-derived foods. Liquid chromatography–tandem mass spectrometry is the most widely used analytical technique for the determination of antibiotic drug residues. This is due to the powerful separation of LC and accurate identification of MS, and LC-MS/MS is more popular in the analysis of veterinary drug residues.

## 1. Introduction

Veterinary antibiotics are extensively applied for therapeutic and prophylactic infections of different animals. By definition, veterinary drugs are any substances applied or administered to any food-producing animals, such as meat or milk-producing animals whether used for therapeutic, prophylactic, or diagnostic purposes [1]. Veterinary drugs can be divided into six classes such as antimicrobials antiparasites, anti-inflammatory drugs, tranquillizers, drugs with growth promotional effect, and others [2].

Antimicrobials are the dominant class of veterinary drugs used after 1950s to treat bacterial infectious diseases in animals. However, after the use of veterinary drugs in food-producing animals, parent compounds and their metabolites may accumulate in the products of animal origin. The normal use of veterinary drugs is acceptable in risk analysis by dietary intake assessment. But, unreasonable use of veterinary drugs due to the lack of scientific knowledge and the blind pursuit of economic benefits by husbandry personnel may lead to the existence of high drug residue in animal-derived food products [3]. The existence of veterinary antibiotic residues in animal products such as milk and meat may cause allergies in humans, and in the extensive and long run may facilitate the development of resistant pathogens. The existence of resistant bacterial strains produces severe health consequences on the human body [4]. These reasons make it important to effectively control antibiotic residues in animal-derived food products, and therefore, regulatory authorities have enacted maximum residue limits (MRLs) for a number of anti-infective agents in milk and meat as described in Table 1. National residue monitoring programs to control veterinary drug residues in different animal-derived food products including meat and milk are compulsory in all nations. Effective veterinary antibiotic monitoring program requires specific, sensitive, and reliable analytical methods that can detect all antibiotic drug residues below regulated levels (maximum residue level) [6]. Various analytical methods have been described to determine antibiotic drug residues in milk and meat, such as chromatographic, immunochemical, and biosensor tests [7]. The methods used in the detection usually depend on the type of antimicrobials targeted, the expected time limitations, selectivity, and its cost. These methods that are used for the measurement of antimicrobial residues may be qualitative and quantitative [4]. Therefore, the objective of this review is to explain the large range of analytical methods developed and used for the determination of antimicrobial drug residues in meat and milk.

## 2. Antimicrobial Drug Residues

Antimicrobial residues are defined as all active ingredients or its metabolites of those drugs or degradation products that remain in animal-derived food products [8]. The use of veterinary antibiotics for food-producing animals may cause the existence of residues in foodstuffs of animal origin like meat and milk [9].

## 3. Risk Factors for Antibiotic Residue Occurrence

Most of the time, livestock producers treat entire groups of livestock animals. This activity unintentionally and unnecessarily can expose healthy animals to antimicrobials. Additionally, many livestock producers administer subtherapeutic doses of veterinary antibiotics to treat infectious diseases, and this will cause the antibiotic residue to enter the human food chain [10]. Animal fecal recycling, where the drug residues can excrete in the feces of treated animals, which will contaminate the feed of untreated animals, can also be the cause of the occurrence of veterinary antibiotic residues [10]. The followings are the major risk factors for antibiotic residue occurrence.

### 3.1. Disease Status

The disease status of an animal can affect the pharmacokinetics of the drugs administered, which can influence the potential for residues [11]. This can occur either when the disease affects the metabolic system (and consequently drug metabolism) or when the presence of infection and/or inflammation causes the drug to accumulate in affected tissues. The changes in liver function by fasciolosis result change in the drug metabolism. The kidney is the most important site of drug excretion. Renal disease usually significantly affects drug excretion (retard drug removal from the body). The systemic clearance and elimination half-life are important parameters referring to the overall rate of elimination (metabolism and excretion). Although most compounds are excreted primarily by the renal, some drugs are partially or completely excreted through the bile. It has been reported that there is an extensive species variation among animals in their general ability to excrete drugs in the bile; for example, chickens are characterized as good biliary excretes, whereas sheep and rabbit are characterized as moderate and poor excretes [11].

### 3.2. Extra-Label Drug Use

Extra-label drug use (ELDU) refers to the use of an approved drug in a manner that is not in accordance with the approved label directions. It occurs when a drug only approved for human use is used in animals when a drug approved for one species of animal is used in another, when a drug is used to treat a condition for which it was not approved, or the use of drugs at levels in excess of recommended doses [12]. For instance, the use of enrofloxacin solution as a topical ear medication (only approved for use as an injection) is the common ELDU in veterinary medicine [13].

### 3.3. Improper Withdrawal Time

Withdrawal time is the time for the residue of toxicological concern to reach a safe level of drug concentration. The withdrawal time can be varied for different veterinary drug products depending on different conditions, such as the type of drug, dosage form, and route of drug administration. Withdrawal time can also be defined as the interval between the last administration of a veterinary drug to the animals under normal conditions of use and the time when a treated animal can be slaughtered for the production of safe foodstuffs [14]. Therefore, failure to wait for the withdrawal period causes the occurrence of residue in foods of animal origin such as meat and milk, which are used for human consumption [10].

## 4. Safety Evaluation of Veterinary Antibiotic Residues

The following parameters are used to evaluate the safety of veterinary antibiotic residues.

### 4.1. Acceptable Daily Intake (ADI)

By definition, acceptable daily intake for a given drug is the amount of a drug that can be ingested every day over a lifetime without appreciable health risks to the consumer [15]. Acceptable daily intake is also defined as a maximum amount of drug residues or chemicals, which can be consumed every day by the most sensitive classes in the population with any outward effects on their health [16].

The acceptable daily intake can be calculated by using a safety or uncertainty factor, which is commonly 100, to the no observed adverse effect level (NOAEL) obtained from the most sensitive test species. The 100-fold safety factor is based on the need to take into account both the variations in species and variations in toxicokinetics and toxicodynamics [17].

(1) $$\begin{array}{c} ADI=Long-term\;NOAEL\;\frac{\left(lowest\;value\right)}{100}\text{.} \end{array}$$

### 4.2. Maximum Residue Limits (MRL)

The term maximum limit for residues of veterinary antibiotics or drugs is the maximum concentration of veterinary drug residues resulting from the use of veterinary drugs legally permitted or recognized as acceptable in animal food products. The concentration of drug residue can be expressed in milligrams/micrograms per kilogram of the commodity (or milligrams/micrograms per liter in the case of a liquid commodity) or ppm/ppb [18]. A residue at or below the stated MRL is considered safe when animal-derived food at that level is consumed daily for a lifetime (Table 1). The MRLs are specified for several animal-derived food products (different edible tissues and other food commodities). When veterinary drugs are used according to the period of treatment and the withholding period specified before slaughter or milking, the concentration of drug residues should be at levels that will not cause an adverse effect on the health of the consumer. Therefore, animals are suitable for food production if the amounts of veterinary antibiotic residues in animal food products are below levels which could cause a health risk for consumers [19].

Nowadays, regulatory bodies have been established for veterinary drugs used in food-producing animals to ensure regular monitoring of veterinary drug residues in livestock products. The regulatory laws can help the government's policies in managing animal-derived food safety, prevention, and control of food safety incidents [18].

## 5. Impact of Antimicrobial Residues

The incidence of veterinary antibiotic residues in animal-derived foods produces a significant health risk for consumers because of the emergence of microbial resistance noticed in recent years [20]. Extensive use of antibiotics might increase the risk of an adverse effect of residues on the customer and the occurrence of antibiotic resistance as well as hypersensitivity reactions in consumers [10]. Therefore, ingenuity in the use of veterinary antibiotics in the manner of preventing animal feed and food contamination is required [16]. The followings are some of the impacts of antibiotic residues.

### 5.1. Antimicrobial Resistance

The emergence of antimicrobial resistance has been observed due to different factors. Some of the factors include repeated use and exposure to sublethal doses of antimicrobials [20]. In addition, the application of animal manure for soil fertilization can be a contemplated contributor to environmental contamination and transmission of antimicrobial drug residues through animal feces. Currently, the development of antimicrobial-resistant bacterial genesis is frequently described owing to the overuse of veterinary antimicrobials all over the world. The utilization of veterinary antimicrobials in food-producing animals causes selection for bacterial resistant to antimicrobials. Administering these antibiotics to humans will result in poor response to treatment during illness [21].

### 5.2. Drug Hypersensitivity

Drug hypersensitivity is defined as an immune arbitrated response to a drug agent in a sensitized patient, and drug allergy is constrained to a reaction mediated by IgE. Allergic reactions to drugs may include anaphylaxis, serum sickness, and cutaneous reaction, and a delayed hypersensitivity response to drugs seems to be more frequently linked with antibiotics, especially penicillin. Penicillin residues in milk could provoke allergic reactions in sensitized individuals [22]. About 10 percent of the human population is considered hypersensitive to an amount of a substance, including penicillin, but in animals, the extent of hypersensitive to the drug is not well known. Certain macrolides might also in exceptional be responsible for liver injuries, triggered by a specific allergic response to macrolide-modified hepatic cells [23].

### 5.3. Teratogenic Effect

The term teratogen applies to a drug or chemical agent that produces a toxic effect on the embryo or foetus during a critical phase of gestation. Consequently, a congenital malformation, which affects the structural and functional integrity of the organism, is produced [24].

## 6. The Extent of Veterinary Antibiotic Residue in Ethiopia

In most African countries, veterinary antibiotics are used to treat infectious diseases or feed domestic animals. The current threat of antimicrobial residue is a major challenge for public health. This challenge faced the human population worldwide, including in Africa [25]. These veterinary antibiotic residues are escalating rapidly, disregarding topographical, biological, or legitimate variations among countries [25, 26].

A study was conducted in Ethiopia for the determination of oxytetracycline and penicillin G residues in milk samples from farms (Nazareth dairy farms). From the total 400 milk samples, 48 milk samples were found to contain oxytetracycline and penicillin G residues.

Further investigation was also carried out in Ethiopia in 2007 to determine the proportion of tetracycline residual levels in cattle. Among the meat samples collected from the three sampling sites (Addis Ababa, Debre Zeit, and Nazareth slaughterhouses), 93.8%, 37.5%, and 82.1% tested positive for oxytetracycline residues, respectively [27].

## 7. Codex and Food Safety System in Ethiopia

By definition, the Codex Alimentarius Commission is the international body that is responsible for the execution of the joint FAO/WHO food standards program [28]. It was established in 1962 by FAO and WHO. The program is aimed at safeguarding the health of customers and facilitating international trade in foods [29].

The Ethiopian National Codex Committee (ENCC) was established under the auspices of the Quality and Standards Authority of Ethiopia (QSAE) in 2003 [28]. The NCC member organizations are Addis Ababa University, the Ministry of Health, the Ministry of Agriculture, the Ministry of Trade and Ministry of Industry, the Ethiopian Public Health Institute and Consumers Association and the Ethiopian Chamber of Commerce, and QSAE [30].

The principal responsibilities of the National Codex Committee are endorsement of recommended Codex standards as Ethiopian standards, representing the country's interest in selected international Codex meetings, detecting priority areas on food safety, expanding fundable projects, and conducting national awareness program on food safety and codex standards [31].

## 8. Analysis of Antibiotic Drug Residues

### 8.1. Sample Pretreatment

The occurrence of antibiotic residues can vary within a single organ, and it is a major factor to consider before sample preparation. For instance, residue differences can occur in the kidney between the medulla and the cortex [32]. Accordingly, it is important to take a characteristic aliquot of the biological sample. This may need the removal of some portions throughout the composite sample to get a representative sample [33]. Homogenisation with a blender is often important to get a homogenous biological sample. Liquid biological samples like milk are generally easier to process than solid samples, and antibiotic residues are more homogenously distributed throughout [33].

### 8.2. Sample Extraction Techniques

Drug residue extraction is the removal of an active agent (antibiotic residue) from a solid (animal tissues and organs) or liquid mixture (from milk) with an extraction solvent. Residues are typically extracted from samples using simple solvent extraction or liquid-liquid extraction (LLE). The extraction technique adopted may depend on the nature of the samples (i.e., liquid or solid) and the physicochemical properties of the residues (polarity and pKa) [33]. The major goal of the sample extraction process is to get a suitable sample for analytical instruments, commonly for chromatographic analysis, that will not contaminate the analytical instrument. The method of biological sample preparation and extraction technique selected is generally dictated by the analytical methods accessible and the physical characteristics of the residues in the process of investigation [33]. The following methods are used to describe the extraction of antibiotic residues in biological samples.

#### 8.2.1. Solvent Extraction Technique

In the solvent extraction method, the biological sample (most of the time, meat) is mixed with the selected extraction medium or solvent. The solvent helps to dissolve veterinary drug residues and other biological extractives. The extractive solvent also promotes the deproteinization of biological samples [30]; most of the time, organic extraction solvents are distinctly important in veterinary drug residue analysis because they enable the extraction of protein-associated veterinary drugs from biological samples. Factors to be considered during the selection of an extraction solvent are the thermodynamic properties and its ability to interact with the analyte [34].

Some organic solvents, such as acetonitrile, methanol, and ethanol, are water miscible and frequently applicable in veterinary drug residue extraction. This is due to the polar behavior of the majority of veterinary antibiotic drugs. Proteins from biological samples are generally not soluble in organic solvents. Therefore, organic solvents help to precipitate proteins, so veterinary drug residues can be left at protein binding sites [35].

For polar antibiotic residues, aqueous extraction solvents can be applied. Assorting the pH of an extraction solvent can increase the polarity of the extraction solvent. Therefore, it may have a greater capacity for solubilizing polar veterinary antibiotic residues. Mineral acids were utilized for the extraction of tetracycline residues since this drug is not acid labile [19]. Popelka et al. [36] used an acidic buffer and heat to extract neomycin from tissues.

#### 8.2.2. Liquid-Liquid Extraction

One of the most useful techniques for residue extraction from the biological matrix is liquid-liquid extraction (LLE) [37]. It is a technique applied for the isolation and extraction of analytes from a mixture using two immiscible extraction solvents [37]. The concept “like dissolves like” works well in the LLE method of antibiotic residue separation from the biological matrix. The capacity to isolate analytes from a mixture using this technique depends upon how differently the compounds in the sample mixture partition themselves between the two immiscible phases (solvents). By carefully choosing an extraction solvent, the analyte of interest (antibiotic residue) is meticulously divided into one of two immiscible or partially miscible phases [34]. LLE separates analytes from interferences by partitioning the sample between two immiscible liquids or phases. First, the component mixture is dissolved in a suitable solvent, and a second solvent that is immiscible with the first solvent is added. Next, the contents are thoroughly mixed (shaking), and the two immiscible solvents are allowed to separate into layers [38]. The less dense solvent will be on the upper layer, while the denser solvent will be on the lower layer. The components of the initial mixture will be distributed amongst the two immiscible solvents as determined by their partition coefficient. The relative solubility that a compound has in two given solvents can provide an estimation of the extent to which a compound will be partitioned between them [39]. A compound that is more soluble in the less dense solvent will preferentially reside in the upper layer. Conversely, a compound more soluble in the denser solvent will preferentially reside in the lower layer. Lastly, the two immiscible layers are separated and transferred, and the component in that solvent is isolated. Generally, after extraction, hydrophilic compounds are seen in the polar aqueous phase, and hydrophobic compounds are found mainly in the organic solvents [38]. Liquid-liquid extraction (LLE) has been exploited as an extraction procedure for aminoglycosides and macrolides from complex matrices. In a method published on determination of the streptomycin and dihydrostreptomycin, milk samples were prepared using LLE [40].

### 8.3. Sample Cleanup Methods

Most biological sample matrices contain endogenous compounds that have a negative impact on the detection of antibiotic residues, so after extraction, different cleanup techniques can be used to remove interferences. Interference is defined as any component of biological samples that can prevent or hinder the process of determining the analyte or drug residues [15]. The followings are important techniques used for the cleanup of drug residues from interferences.

#### 8.3.1. Dispersive-Solid Phase Extraction (DSPE)

It is a cleanup method that requires the mixing of sorbent with the sample, which has been pre-extracted using proper extraction techniques. Proper sorbents adsorb matrix coextractives onto their surface, leaving analytes of interest in the solvent [41]. In the process, magnesium sulfate (MgSO_4_) can be added to get extra cleanup by withdrawing residual water and some other components *via* chelation [41]. Subsequently, the mixture can be centrifuged, and the resulting supernatant or filtrate can be analyzed directly or can be subjected to a concentration and solvent exchange step [42]. DSPE is an extremely rapid, simple, and cheap process that provides high recovery and reproducibility for many liquid chromatography and gas chromatography-amenable analytes [42].

#### 8.3.2. Immunoaffinity Column Chromatography

Nowadays, immunochemical methods are mostly applied for the separation of antibiotic residues from biological materials. This method gives high specificity, sensitivity, and sample throughput [43]. In this technique, antibodies against the analytes or residue of interest will be immobilized on the surface of a solid sorbent support that is packed into a syringe barrel. For the development of the immunoaffinity column method, different parameters may be adjusted to attain ideal separations. Some of the parameters include the properties of the sorbent, the integrating mechanism for immobilization of the antibody on the surface of the sorbent, and the property of the antibody. Optimal sample loading, proper cleaning, and elution techniques should be determined after the preparation of the immunoaffinity column. The effectiveness of the technique depends on the functionality of the antibody to bind the residue or analyte of interest. For better antibody-antigen interaction, it is necessary to work under optimal conditions, which are as close as possible to physiological conditions. Such characteristics limit the application of this technique to polar veterinary antibiotics. Maximal heat, pH, and organic solvent content may cause the denaturation of antibodies on the solid support. Throughout sample loading, some conditions must behave to the establishment of the antigen and antibody complex. The formed antigen-antibody complex should not be overblown by washing solvents and elution conditions, which are essential for the dissociation of the antigen-antibody complex [44]. Additionally, the dissociation of antibody/antigen complex must ideally be reversible. Therefore, the antigen-antibody complex can be comfortably reformed, which helps reuse the immunoaffinity column. Hou et al. [45] used immunoaffinity chromatography cleanup for the simultaneous analysis of avermectins in bovine tissues by the LC-MS-MS method [45].

## 9. Screening Methods

Several tests have been described for the screening of antimicrobial residues in various biological samples. Bio-based screening methods applied for the detection of antimicrobials in animal-derived food products have been reviewed [36, 46, 47]. The most frequently used bio-based screening methods for antimicrobials are microbiological inhibition assays, immunoassays, and biosensor tests [48].

**Figure 1:**
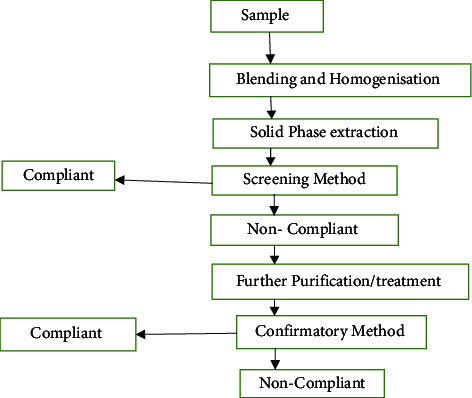
Example of typical processes for the determination of a given analyte in a meat sample.

In the process of screening methods, compliant samples are accepted, and suspected noncompliant samples have to be rechecked and confirmed using other confirmatory methods. A scheme of the typical screening analysis procedure is shown in Figure 1. In antibiotic residue determination, high-throughput methods with low cost and the ability to identify an analyte or class of analytes at the level of interest are needed [49]. In the event, antibiotics have a maximum residue limit, and the screening analytical method should be able to identify the residue under the maximum limit. The screening analytical methods must also avoid false negative results because they will be considered as compliant samples and will not be analyzed or determined by confirmatory analytical methods. Additionally, the screening analytical method must not give an excessive number of falsely noncompliant samples that will be later confirmed as compliant, despite the extra cost and time involved [50].

The followings are the important terminologies used to describe the screening analytical methods and to evaluate antibiotic residues in milk and meat.

### 9.1. Microbiological Inhibition Assays

Microbiological inhibition assays are one of the most widely used screening analytical methods. The principle of this technique is based on a reaction between bacteria and antimicrobials that are present in biological samples. Various biological tests were expanded to screen various antibiotic residues from animal-derived food products [36]. There are two most common formats for microbiological inhibition assays, such as the tube and plate tests [51].

The tube test of a microbiological assay comprises of a growth medium inoculated with a bacterium, supplemented with a pH or redox indicator. Then, biological samples are added to the tube, and if there are no particular antimicrobials present in the biological sample, the bacteria begin to grow and produce acid, which will cause a detectable color change. Conversely, if antimicrobials are present in the biological sample that inhibits bacterial growth, no color change will occur in the tube [52].

The plate microbiological test consists of a layer of nutrient agar inoculated with bacteria, and the biological samples are brought onto the surface. If there is no specific antimicrobials are present in the biological sample, the bacteria begin to grow throughout the plate. If a specific antibiotic is present in the biological sample (meat or milk), no bacterial growth will take place on the sample that can be observed from the bacterial-free inhibition zone [51].

Now, microbiological inhibition tests are available in kits that can test a lot of samples quickly. This is called “high productivity.” Microbiological tests need restricted laboratory capacity to make certain reproducible situation of application. Microbiological tests are extensively used to perform antibiotic residue control [53].

The advantage of microbiological inhibition assays compared to immunoassays and instrumental analytical methods is that microbiological tests can detect any antibiotic residues that show antibacterial activity [54]. Moreover, these tests have the potential to cover the entire antibiotic spectrum within a single test [53]. The limitation of these techniques is their lack of selectivity, especially the tube microbiological inhibition test, relatively high detection limits, and the long bacterial incubation time. Consequently, microbiological inhibition assays are not suitable for the detection of banned antibiotic compounds like chloramphenicol [51].

### 9.2. Immunological Techniques

Antigen-antibody interaction has been used for many years to identify a wide variety of food constituents, including substances responsible for adulteration and contamination [55]. The interaction of antigen and antibody is very specific and useful for the detection of veterinary drug residues in animal-derived food products. The most widely used method comprises of the enzyme-linked immunosorbent assay (ELISA). Detection sensitivity depends on the strength of the signals during the reaction [56]. The ELISA-based detection system is usually based on enzyme-labeled reagents. There are various formats for the enzyme-linked immunosorbent assay (ELISA) technique. The first form of the ELISA technique is sandwich ELISA tests; in this technique, a primary antibody is bound to the plate well. Then, the antigen of the sample extract was added to the well complexes with the bound antibody and remains bound to the plate after washing. Then, a secondary antibody, which is labeled with an enzyme such as peroxidase, is added to the well followed by additional washing of the well. The quantity of conjugate bound to the plate is detected after incubation with a specific substrate [32]. The color is developed during incubation and measured with a microplate reader, which is proportional to the amount of analyte in the sample [32].

The second type of ELISA technique is direct competitive ELISA; in this technique, a primary antibody is coated onto the plate wells and incubated with the sample extract containing the antigens. After equilibrium is reached, an enzyme-labeled antigen can be added. This conjugate will bind to the free binding sites of the primary antibody. Thus, the more antigen in the sample (biological sample in this case), the lower the amount of enzyme-labeled antigen bound will be formed. Then, the appropriate specific substrate is added, and the plate is incubated for color development. In this case, there is an inverse relationship between the color developed and the concentration of the analyte in the sample [57].

The ELISA technique is an extensively used and specific test for the screening of veterinary antimicrobial residues in animal-derived food products. The competitive ELISA technique is frequently applied for the quantitative determination of antibiotic residues in meat and milk [57, 58]. Gaurav et al. [59] reported that tetracycline residues were detected in milk by competitive ELISA. From 133 cattle milk samples, 18 samples were found to be contaminated with tetracycline. The concentration of tetracycline residues in milk samples was found to be in the range 16–134.5 *μ*g/l. According to the report, three samples exceeded the maximum recommended tetracycline antibiotic residue levels (MRLs). Sultan [60] reported that enrofloxacin residues in liver sample of poultry, sheep, and cattle collected from slaughter house Iraq. Out of 30 samples from each species, 17 poultry samples, 8 cattle samples, and 5 sheep samples exceeded the maximum residue limits. The concentration of enrofloxacin in liver sample of poultry, cattle, and sheep was 10–10690, 30–3610, and 20–1320 *μ*g/kg, respectively.

### 9.3. Biosensors

Biosensor is one of the screening analytical methods for veterinary drug residue analysis. Various types of biosensors (such as immunobiosensor, bacterial biosensor, optical biosensor) have been developed to determine antimicrobial drug residues. Biosensors employ biological molecules, such as enzymes or antibodies, which are efficient for recognizing particular targeted analytes or residues. In the detection process, the molecules are paired to a transducer, which response to the reaction between the residue and the bound biological molecule. The resulting biochemical alert is observed optically or changed to an electronic signal, which is additionally clarified by a suitable instrument. Biosensor tests are capable of identifying concurrent multiclass antibiotics and pesticides in biological samples at the same time. As some authors described, there is no need for sample cleanup for biosensor analytical technique [61]. A report from Möhrle et al. [62] showed that the biosensor method was used for the screening of macrolide antibiotics. Using an electrochemical biosensor, Ferrini et al. [63] determined *β*-lactams in milk samples by means of CO_2_ measurement. The production of CO_2_ was related to the microbial growth of the test microorganism, and the presence of *β*-lactams in milk inhibits the microbial growth.

### 9.4. High-Performance Thin-Layer Chromatography (HPTLC)

HPTLC analytical technique allows the qualitative and quantitative determination of multidrug residues in animal-derived food products (meat and milk), but nowdays, its applicability has rapidly decreased due to the development of other advanced techniques like high-performance liquid chromatography (HPLC) [64]. Reported uses of HPTLC applied to meat include the determination of veterinary drug residues such as clenbuterol and other agonists [65], nitroimidazole and sulphonamides, and thyreostatic drugs [66]. Bartolucci et al. [67] presented a method using a TLC plate precoated with silica gel and eluting chemicals with 0.5 ml methanol-acetic acid-acetone (1 : 5 : 94, v/v/v) to analyze the sulfamethoxazole residues in milk. Reimer and Suarez [68] developed a TLC method with a high-performance TLC plate eluting with ethyl acetate-n-butanol-methanol-aqueous ammonia (35 : 45 : 15 : 2, v/v/v/v) to analyze sulphonamides in salmon muscle tissue. During the detection process, the HPTLC plates are sprayed with a proper chromogenic reagent or viewed under UV light for visualization of compounds. Detection by fluorescence is also applied. Quantitative analysis is achieved by measuring the relative intensity of the spot of the sample *vs* that of the internal standard by scanning densitometry [66].

### 9.5. High-Performance Liquid Chromatography (HPLC)

High-performance liquid chromatography is a separation analytical technique. The principles of HPLC involve the injection of a small volume of liquid samples into a tube (column) packed with tiny particles called the stationary phase. Individual components of the sample are moved down the packed column with a liquid (mobile phase) forced through the column by high pressure delivered by a pump. The sample components are separated from one another by the column packing that involves various chemical interactions between the molecules and the packing stationary phase. The separated components are detected at the exit of the column by a flow-through device (detector) that measures their amount. An output from this detector is called a liquid chromatogram [69].

High-performance liquid chromatography is one of the most powerful analytical instruments in pharmaceutical analysis and analytical chemistry. HPLC has the ability to separate, identify, and quantitate the compounds that are present in any sample that can be dissolved in a liquid. Compounds at very low concentrations (as low as parts per trillion) may be easily identified by this technique. HPLC can be and has been applied to just about any sample, such as pharmaceuticals, food, nutraceuticals, cosmetics, environmental matrices, and forensic samples [69].

In the field of analyzing drug residues in biological samples from animals, HPLC is being used more and more every day. HPLC has different mobile phases, a vast library of column packings, and various modes of operations [70]. HPLC was used for veterinary drug residue determination of oxytetracycline and penicillin G in milk in Ethiopia, specifically samples collected from Nazareth dairy farms [71]. From 400 milk samples, 48 milk samples were found to contain oxytetracycline and penicillin G in the range of 45–192 *μ*g/l and 0–28 *μ*g/l, respectively.

HPLC technique was also used for the determination of sulphanilamide, tetracycline, streptomycin, and ciprofloxacin In South Africa. A total of 150 samples of raw meat from sales points were analyzed, and the concentration ranges of 20.7–82.1, 41.8–320.8, 65.2–952.2, and 32.8–95.6 for sulphanilamide, tetracycline, streptomycin, and ciprofloxacin, respectively [72]. A study was also conducted in Iran to determine the residues of tetracycle groups (tetracycline, oxytetracycline, and chlortetracycline) from cattle tissue and organs, by using a high-performance liquid chromatography technique. The tetracycline concentrations in the triceps muscle, gluteal muscle, diaphragm, kidney, and liver were 176.3, 405.3, 96.8, 672.4, and 651.3 ng/g, respectively. The concentrations of tetracyclines were higher in liver and kidney samples compared to other samples [73] and were higher in cured meat products [74].

## 10. Confirmatory Methods for Antibiotic Residues

Confirmatory methods, mainly based on liquid chromatography combined with tandem mass spectrometry (LC-MS^2^), are required for unequivocal identification and, if necessary, quantification of the analyte of interest. Only after a confirmatory analysis, a suspected contaminated sample will be declared noncompliant. However, Commission Decision 2002/657/EC still accepts detection techniques such as diode-array (DAD) or fluorimetric detection (FLD) as possible confirmatory techniques; nevertheless, from the practical point of view, confirmation of antibacterial residues in food is performed by LC-MS techniques since they can provide information about the chemical structure of the analyte [75].

Confirmatory analytical methods or techniques for determining veterinary drug residues or contaminants should give real information about the chemical structure of the residue or analytes. Methods that only use chromatographic analysis and do not use spectrometric detection are not good enough to be used as confirmatory methods on their own. However, if a single technique lacks sufficient specificity, the desired specificity may be achieved by analytical procedures consisting of suitable combinations of cleanup, chromatographic separations, and spectrometric identification [64].

LC-MS is the most commonly employed method for the determination of veterinary drug residues in animal-derived foods products [76]. The LC-MS technique uses LC as the separation system and MS as the detection system [77]. In order to quickly separate and identify numerous residues, it combines the high selectivity, high sensitivity, and relative molecular mass information of MS with the great separation capacity of chromatography for complicated materials.

LC-MS technique was applied for the determination of macrolides [78], sulphonamides [79], tetracyclines [80, 81] penicillins [82], quinolones and fluoroquinolones [83], and aminoglycosides [84] in the food of animal origin. High-resolution liquid chromatography combined with TOF-MS was used for the multiresidue determination of about 100 veterinary drugs in egg, fish, and meat [85]. LC/MS method was also developed and used for the identification and quantification of 30 antibiotics from four different chemical classes (sulphonamides, tetracyclines, quinolones, and beta-lactams) in Lebanon (Table 2). Out of 80 chicken muscle samples collected, 77.5% of samples were contaminated with antibiotic residues, out of which 53.75% were exposed to co-occurrence of multidrug residues [72].

## 11. Conclusion

The repeated application of veterinary drugs to animals resulted in the occurrence of residues at various concentration levels in animal-derived food products. In particular, antibiotic residues in the dairy and meat industries may result in antibiotic resistance, which has an extensive public health consequence. The harmful effects of drug residues residing in animal-derived food products may also induce carcinogenic and mutagenic effects and lead to the condition of antimicrobial allergy in individuals who consume animal-derived food products. Accordingly, it is important to effectively control antibiotic residues in animal-derived food products. Effective residue monitoring requires specific, sensitive, and reliable analytical methods that can identify all veterinary drug residues under-regulated levels (MRL). In this review, sample extraction methods and analytical techniques for the determination of veterinary drug residues are summarized. For sample extraction techniques, LLE is widely used for the extraction of residue from biological samples. Solvent extraction especially organic solvent extraction is also applicable for residue extraction. The two types of analytical methods such as screening and confirmatory methods are discussed. Screening methods comprise microbiological, immunological, biosensor, thin-layer chromatography, and high-performance liquid chromatography. Liquid chromatography–tandem mass spectrometry is the most widely used confirmatory analytical technique for the determination of antibiotic residue in animal-derived food products. To sum up, determination methods for veterinary drug residues are developing toward high speed, high sensitivity, high throughput, and multiple residues.

## Figures and Tables

**Table 1:** Maximum antibiotic drug residue limits for commonly used antimicrobials in food staffs of animal derived (from annex I [5]).

Antimicrobial	Muscle (*μ*g/kg)	Liver (*μ*g/kg)	Kidney (*μ*g/kg)	Fat (*μ*g/kg)	Cow milk (*μ*g/kg)
Amoxicillin	50	50	50	50	4
Benzyl penicillin	50	50	50	50	4
Chlortetracycline/oxytetracycline/tetracycline	100	300	600	—	100
Streptomycin/dihydrostreptomycin	600	600	1000	600	200
Closantel	1000	1000	3000	3000	—
Spiramycin	200	300	300	300	—
Cefquinome	50	100	200	50	20
Tilmicosin	50	1000	1000	50	50
Tylosin	100	100	100	100	50

**Table 2:** Antibiotic residues in different animal products.

Name of the antibiotic	Matrix	Extraction technique	Purification technique	Detection system	Recovery (%)	Reference
Tetracycline	Muscle	MSPD	Elution solvent: H_2_O (70°C)	LC-MS/MS	99–103	[86]
Tetracycline	Milk	LLE	SPE (oasis HLB)	LC-MS/MS	74–101	[87]
Tetracycline	Porcine kidney	MIPs	Elution solvent: MeOH : 1 M KOH (9 : 1, v/v)	HPLC-UV		[88]
Sulfonamide	Milk	LLE	Ultrafiltration	LC-MS/MS	90–125	[89]
Sulfonamide	Muscle	LLE	LLP (H_2_O: EtOAc)	UPLC-MS/MS	68–114	[90]
Quinolones	Bovine tissues	MSPD (sand)	Elution solvent: H_2_O (100°C)	LC-MS/MS	87–109	[91]
Quinolones	Milk	MSPD (sand)	Elution solvent: H_2_O (100°C)	LC-MS/MS	93–110	[77]
Quinolones	Eggs and tissue	MIPs	Elution solvent: ACN : TFA (99 : 1, v/v)	HPLC-FL	86–105	[92]
Aminoglycosides	Milk	MSPD (sand)	PLE	LC-MS/MS	70–92	[93]
Aminoglycosides	Muscle, liver	LSE	SPE (WCX)	LC-MS/MS	61–116	[94]
*β*-Lactams	Bovine kidney	LSE	DSPE (C_18_)	LC-MS/MS	58–75	[76]
*β*-Lactams	Milk	LLE	LLP	HPLC-UV	94–103	[95]
*β*-Lactams	Muscle	LSE	Ion-exchange SPE	LC-MS/MS	87–103	[96]

## Data Availability

The data supporting the current study are available from the corresponding author upon request.
